# Bis(2,2′,2′′-nitrilo­triacetamide-κ^3^ *O*,*N*,*O*′)cobalt(II) dinitrate tetra­hydrate

**DOI:** 10.1107/S1600536813012968

**Published:** 2013-05-18

**Authors:** Jing-Wen Ran, Jun Pei

**Affiliations:** a College of Chemical Engineering, Huanggang Normal University, Huanggang 438000, People’s Republic of China; b College of Education Science and Technology, Huanggang Normal University, Huanggang 438000, People’s Republic of China

## Abstract

In the centrosymmetric title compound, [Co(C_6_H_12_N_4_O_3_)_2_](NO_3_)_2_·4H_2_O, the Co^II^ ion, lying on an inversion center, is *O*,*N*,*O*′-chelated by two nitrilo­triacetamide mol­ecules, forming a distorted octa­hedral geometry. In the crystal, extensive O—H⋯O and N—H⋯O hydrogen bonds link the complex cations, nitrate anions and lattice water mol­ecules into a three-dimensional network.

## Related literature

For related structures, see: Kumari *et al.* (2012 ▶). For the synthesis of the ligand, see: Smith *et al.* (1995 ▶).
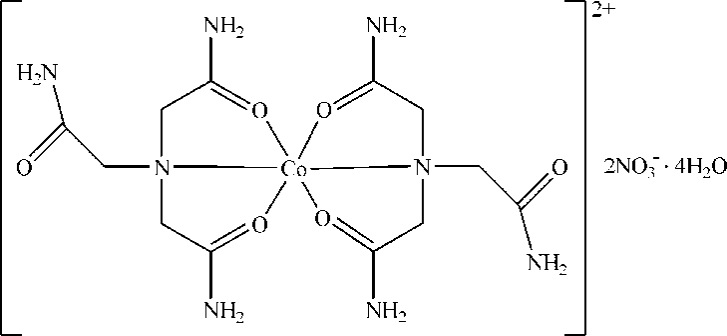


## Experimental

### 

#### Crystal data

[Co(C_6_H_12_N_4_O_3_)_2_](NO_3_)_2_·4H_2_O
*M* *_r_* = 631.41
Triclinic, 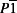

*a* = 8.4910 (17) Å
*b* = 9.1410 (18) Å
*c* = 9.2580 (19) Å
α = 91.55 (3)°
β = 96.03 (3)°
γ = 110.68 (3)°
*V* = 667.0 (2) Å^3^
*Z* = 1
Mo *K*α radiation
μ = 0.73 mm^−1^
*T* = 293 K
0.36 × 0.32 × 0.25 mm

#### Data collection

Siemens P4 diffractometer
Absorption correction: ψ scan (*XSCANS*; Siemens, 1994 ▶) *T*_min_ = 0.779, *T*_max_ = 0.838
3692 measured reflections
2301 independent reflections
2185 reflections with *I* > 2σ(*I*)
*R*_int_ = 0.016
2 standard reflections every 150 reflections intensity decay: none

#### Refinement

*R*[*F* ^2^ > 2σ(*F* ^2^)] = 0.028
*wR*(*F* ^2^) = 0.080
*S* = 1.05
2301 reflections
191 parameters
6 restraints
H atoms treated by a mixture of independent and constrained refinement
Δρ_max_ = 0.32 e Å^−3^
Δρ_min_ = −0.22 e Å^−3^

### 

Data collection: *XSCANS* (Siemens, 1994 ▶); cell refinement: *XSCANS*; data reduction: *XSCANS*; program(s) used to solve structure: *SHELXS97* (Sheldrick, 2008 ▶); program(s) used to refine structure: *SHELXL97* (Sheldrick, 2008 ▶); molecular graphics: *SHELXTL* (Sheldrick, 2008 ▶); software used to prepare material for publication: *SHELXTL*.

## Supplementary Material

Crystal structure: contains datablock(s) I, global. DOI: 10.1107/S1600536813012968/hy2611sup1.cif

Structure factors: contains datablock(s) I. DOI: 10.1107/S1600536813012968/hy2611Isup2.hkl

Additional supplementary materials:  crystallographic information; 3D view; checkCIF report

## Figures and Tables

**Table 1:** Hydrogen-bond geometry (Å, °)

*D*—H⋯*A*	*D*—H	H⋯*A*	*D*⋯*A*	*D*—H⋯*A*
N2—H2*A*⋯O6^i^	0.86	2.26	3.026 (3)	149
N2—H2*B*⋯O3^ii^	0.86	1.96	2.816 (2)	173
N3—H3*C*⋯O8^iii^	0.86	2.11	2.958 (3)	168
N3—H3*D*⋯O5^ii^	0.86	2.16	3.001 (3)	166
N4—H4*A*⋯O7^iv^	0.86	2.20	2.992 (3)	152
N4—H4*B*⋯O7	0.86	2.30	3.045 (3)	145
O7—H7*A*⋯O6	0.88 (2)	2.05 (2)	2.877 (3)	156 (3)
O7—H7*B*⋯O8^v^	0.87 (2)	1.96 (2)	2.826 (3)	176 (3)
O8—H8*A*⋯O1^i^	0.85 (2)	2.14 (2)	2.976 (2)	166 (3)
O8—H8*B*⋯O6	0.85 (2)	2.16 (2)	2.980 (3)	161 (3)
O8—H8*B*⋯O5	0.85 (2)	2.42 (3)	3.078 (3)	134 (3)

Symmetry codes: (i) 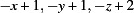; (ii) 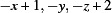; (iii) 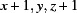; (iv) 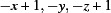; (v) 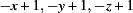.

**Fig. 1.**
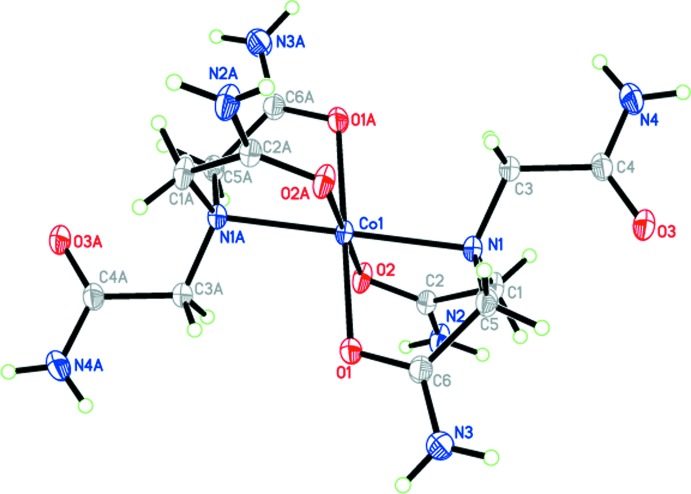
The molecular structure of the complex cation in the title compound. Displacement ellipsoids are drawn at the 30% probability level. [Symmetry code: (A) 2-x, 1-y, 2-z.]

**Fig. 2.**
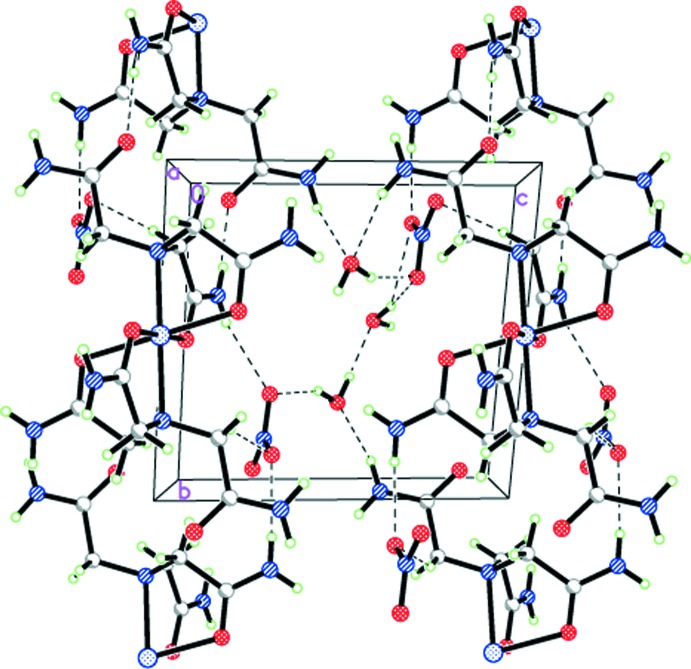
The packing diagram for the title compound, viewed down the a axis, with hydrogen bonds drawn as dashed lines.

## supplementary crystallographic information

### Comment

Transition metal compounds have been of great interest for many years. They are very important in the development of coordination chemistry. As an extension of work on the structural characterization of Co compounds, we report here the crystal structure of a new mononuclear cobalt(II) compound.

The title compound consists of a [Co(NTA)_2_]^2+^ cation (NTA = nitrilotriacetamide), two nitrate anions and four solvent water molecules. The Co^II^ atom has a distorted octahedral coordination environment (Fig. 1), which is centrosymmetric as the Co^II^ atom occupies an inversion center. In the equatorial plane, the Co—N1 distance is 2.1696 (16) Å and the Co—O1 distance is 2.1057 (14) Å. The axial Co—O2 bond is appreciably shortened, which is 2.0329 (14) Å. In the crystal, extensive O—H···O and N—H···O hydrogen bonds (Table 1) link the complex cations, nitrate anions and lattice water molecules into a three-dimensional network (Fig. 2).

### Experimental

The ligand was prepared according to the literature method (Smith *et al.*, 1995). The title compound was synthesized by adding water solution of Co(NO_3_)_2_.6H_2_O (291 mg, 2 mmol) to a solution of the ligand (752 mg, 4 mmol) in methanol/water (v/v 3:1, 20 ml). The mixture was stirred for 30 min at room temperature. The solution was filtered and the filtrate was allowed to stand in air for 1 week, and pink crystals were formed at the bottom of the vessel on slow evaporation of the solvent at room temperature (yield: 30%).

### Refinement

H atoms on C and N atoms were positioned geometrically and refined as riding atoms, with C—H = 0.97, N—H = 0.86 Å, and with *U*_iso_(H) = 1.2*U*_eq_(C,N). The water H atoms were located from a difference Fourier map and refined with restraints of O—H = 0.86 (1) Å and *U*_iso_(H) = 1.5*U*_eq_(O).

### Crystal data

**Table:**

[Co(C_6_H_12_N_4_O_3_)_2_](NO_3_)_2_·4H_2_O	*Z* = 1
*M**_r_* = 631.41	*F*(000) = 329
Triclinic, *P*1	*D*_x_ = 1.572 Mg m^−^^3^
Hall symbol: -P 1	Mo *K*α radiation, λ = 0.71073 Å
*a* = 8.4910 (17) Å	Cell parameters from 4804 reflections
*b* = 9.1410 (18) Å	θ = 2.4–28.3°
*c* = 9.2580 (19) Å	µ = 0.73 mm^−^^1^
α = 91.55 (3)°	*T* = 293 K
β = 96.03 (3)°	Block, pink
γ = 110.68 (3)°	0.36 × 0.32 × 0.25 mm
*V* = 667.0 (2) Å^3^	

### Data collection

**Table:**

Siemens P4 diffractometer	2185 reflections with *I* > 2σ(*I*)
Radiation source: fine-focus sealed tube	*R*_int_ = 0.016
Graphite monochromator	θ_max_ = 25.0°, θ_min_ = 2.2°
ω scans	*h* = −8→10
Absorption correction: ψ scan (*XSCANS*; Siemens, 1994)	*k* = −10→6
*T*_min_ = 0.779, *T*_max_ = 0.838	*l* = −11→11
3692 measured reflections	2 standard reflections every 150 reflections
2301 independent reflections	intensity decay: none

### Refinement

**Table:**

Refinement on *F*^2^	Secondary atom site location: difference Fourier map
Least-squares matrix: full	Hydrogen site location: inferred from neighbouring sites
*R*[*F*^2^ > 2σ(*F*^2^)] = 0.028	H atoms treated by a mixture of independent and constrained refinement
*wR*(*F*^2^) = 0.080	*w* = 1/[σ^2^(*F*_o_^2^) + (0.0402*P*)^2^ + 0.3421*P*] where *P* = (*F*_o_^2^ + 2*F*_c_^2^)/3
*S* = 1.05	(Δ/σ)_max_ = 0.001
2301 reflections	Δρ_max_ = 0.32 e Å^−^^3^
191 parameters	Δρ_min_ = −0.22 e Å^−^^3^
6 restraints	Extinction correction: *SHELXL97* (Sheldrick, 2008), Fc^*^=kFc[1+0.001xFc^2^λ^3^/sin(2θ)]^-1/4^
Primary atom site location: structure-invariant direct methods	Extinction coefficient: 0.036 (4)

### Special details

**Table:**

Geometry. All e.s.d.'s (except the e.s.d. in the dihedral angle between two l.s. planes) are estimated using the full covariance matrix. The cell e.s.d.'s are taken into account individually in the estimation of e.s.d.'s in distances, angles and torsion angles; correlations between e.s.d.'s in cell parameters are only used when they are defined by crystal symmetry. An approximate (isotropic) treatment of cell e.s.d.'s is used for estimating e.s.d.'s involving l.s. planes.
Refinement. Refinement of *F*^2^ against ALL reflections. The weighted *R*-factor *wR* and goodness of fit *S* are based on *F*^2^, conventional *R*-factors *R* are based on *F*, with *F* set to zero for negative *F*^2^. The threshold expression of *F*^2^ > σ(*F*^2^) is used only for calculating *R*-factors(gt) *etc*. and is not relevant to the choice of reflections for refinement. *R*-factors based on *F*^2^ are statistically about twice as large as those based on *F*, and *R*- factors based on ALL data will be even larger.

### Fractional atomic coordinates and isotropic or equivalent isotropic displacement parameters (Å^2^)

**Table:**

	*x*	*y*	*z*	*U*_iso_*/*U*_eq_	
Co1	1.0000	0.5000	1.0000	0.02488 (14)	
O1	1.04636 (18)	0.42993 (15)	1.21011 (14)	0.0357 (3)	
N1	0.84838 (18)	0.25174 (17)	0.97367 (17)	0.0255 (3)	
O2	0.77965 (17)	0.51316 (15)	1.05517 (16)	0.0358 (3)	
N2	0.5284 (2)	0.37589 (19)	1.1249 (2)	0.0385 (4)	
H2A	0.5140	0.4602	1.1531	0.046*	
H2B	0.4520	0.2860	1.1335	0.046*	
C2	0.6662 (2)	0.3843 (2)	1.0691 (2)	0.0279 (4)	
C4	0.7310 (2)	0.0285 (2)	0.7776 (2)	0.0309 (4)	
C1	0.6803 (2)	0.2307 (2)	1.0199 (2)	0.0344 (5)	
H1A	0.6607	0.1616	1.0990	0.041*	
H1B	0.5929	0.1810	0.9393	0.041*	
C5	0.9455 (3)	0.1821 (2)	1.0737 (2)	0.0311 (4)	
H5A	1.0417	0.1752	1.0298	0.037*	
H5B	0.8741	0.0772	1.0931	0.037*	
C6	1.0065 (2)	0.2848 (2)	1.2144 (2)	0.0320 (4)	
C3	0.8369 (3)	0.2003 (2)	0.8196 (2)	0.0319 (4)	
H3A	0.9508	0.2204	0.7957	0.038*	
H3B	0.7894	0.2642	0.7604	0.038*	
O3	0.7098 (2)	−0.07085 (16)	0.86712 (17)	0.0436 (4)	
N3	1.0164 (3)	0.2180 (2)	1.3353 (2)	0.0498 (5)	
H3C	1.0514	0.2739	1.4165	0.060*	
H3D	0.9878	0.1178	1.3342	0.060*	
N4	0.6714 (3)	−0.0027 (2)	0.6381 (2)	0.0481 (5)	
H4A	0.6138	−0.0976	0.6053	0.058*	
H4B	0.6903	0.0720	0.5802	0.058*	
O4	0.3650 (3)	0.0756 (2)	0.7431 (3)	0.0729 (6)	
N5	0.3013 (3)	0.1744 (2)	0.7124 (2)	0.0490 (5)	
O6	0.3922 (3)	0.3140 (2)	0.7051 (3)	0.0790 (7)	
O5	0.1443 (3)	0.1328 (2)	0.6828 (3)	0.0753 (6)	
O8	0.1387 (2)	0.4513 (2)	0.58788 (17)	0.0494 (4)	
O7	0.6172 (3)	0.2799 (2)	0.5059 (2)	0.0606 (5)	
H8A	0.072 (4)	0.470 (4)	0.642 (3)	0.091*	
H8B	0.195 (4)	0.405 (4)	0.637 (3)	0.091*	
H7A	0.566 (4)	0.319 (4)	0.566 (3)	0.091*	
H7B	0.689 (4)	0.364 (3)	0.475 (4)	0.091*	

### Atomic displacement parameters (Å^2^)

**Table:**

	*U*^11^	*U*^22^	*U*^33^	*U*^12^	*U*^13^	*U*^23^
Co1	0.0248 (2)	0.0160 (2)	0.0289 (2)	0.00148 (14)	0.00284 (14)	0.00066 (13)
O1	0.0466 (8)	0.0217 (7)	0.0305 (7)	0.0042 (6)	−0.0021 (6)	0.0001 (5)
N1	0.0247 (8)	0.0181 (7)	0.0304 (8)	0.0039 (6)	0.0024 (6)	−0.0002 (6)
O2	0.0316 (7)	0.0186 (7)	0.0550 (9)	0.0044 (6)	0.0126 (6)	0.0028 (6)
N2	0.0346 (9)	0.0217 (8)	0.0584 (11)	0.0066 (7)	0.0156 (8)	0.0010 (8)
C2	0.0262 (9)	0.0220 (9)	0.0330 (10)	0.0064 (8)	0.0018 (7)	0.0018 (7)
C4	0.0296 (10)	0.0236 (9)	0.0357 (11)	0.0052 (8)	0.0043 (8)	−0.0038 (8)
C1	0.0269 (10)	0.0204 (9)	0.0521 (12)	0.0030 (8)	0.0085 (9)	−0.0014 (8)
C5	0.0367 (10)	0.0199 (9)	0.0347 (10)	0.0091 (8)	−0.0002 (8)	0.0008 (7)
C6	0.0331 (10)	0.0254 (10)	0.0334 (10)	0.0069 (8)	−0.0013 (8)	0.0026 (8)
C3	0.0374 (11)	0.0220 (9)	0.0291 (10)	0.0025 (8)	0.0027 (8)	0.0003 (7)
O3	0.0528 (9)	0.0226 (7)	0.0426 (9)	−0.0009 (6)	0.0011 (7)	0.0011 (6)
N3	0.0765 (14)	0.0299 (9)	0.0343 (10)	0.0119 (9)	−0.0060 (9)	0.0041 (8)
N4	0.0604 (12)	0.0313 (9)	0.0387 (10)	0.0034 (9)	−0.0061 (9)	−0.0075 (8)
O4	0.0603 (12)	0.0588 (12)	0.1021 (17)	0.0247 (10)	0.0046 (11)	0.0217 (11)
N5	0.0592 (13)	0.0371 (11)	0.0523 (12)	0.0154 (10)	0.0204 (10)	0.0052 (9)
O6	0.0978 (16)	0.0309 (9)	0.0995 (16)	0.0039 (10)	0.0445 (13)	−0.0050 (10)
O5	0.0557 (12)	0.0608 (12)	0.1194 (19)	0.0294 (10)	0.0200 (12)	0.0227 (12)
O8	0.0611 (11)	0.0471 (10)	0.0394 (9)	0.0186 (8)	0.0064 (8)	0.0022 (7)
O7	0.0692 (13)	0.0430 (10)	0.0559 (11)	0.0022 (9)	0.0120 (9)	0.0010 (8)

### Geometric parameters (Å, º)

**Table:**

Co1—O2	2.0329 (14)	C5—C6	1.516 (3)
Co1—O1	2.1057 (14)	C5—H5A	0.9700
Co1—N1	2.1696 (16)	C5—H5B	0.9700
O1—C6	1.250 (2)	C6—N3	1.299 (3)
N1—C3	1.472 (2)	C3—H3A	0.9700
N1—C5	1.478 (2)	C3—H3B	0.9700
N1—C1	1.482 (2)	N3—H3C	0.8600
O2—C2	1.251 (2)	N3—H3D	0.8600
N2—C2	1.307 (3)	N4—H4A	0.8600
N2—H2A	0.8600	N4—H4B	0.8600
N2—H2B	0.8600	O4—N5	1.231 (3)
C2—C1	1.513 (3)	N5—O6	1.245 (3)
C4—O3	1.225 (3)	N5—O5	1.247 (3)
C4—N4	1.321 (3)	O8—H8A	0.85 (2)
C4—C3	1.524 (3)	O8—H8B	0.85 (2)
C1—H1A	0.9700	O7—H7A	0.88 (2)
C1—H1B	0.9700	O7—H7B	0.87 (2)
			
O2^i^—Co1—O2	180.0	N1—C1—C2	112.43 (15)
O2^i^—Co1—O1	91.39 (7)	N1—C1—H1A	109.1
O2—Co1—O1	88.61 (7)	C2—C1—H1A	109.1
O2^i^—Co1—O1^i^	88.61 (7)	N1—C1—H1B	109.1
O2—Co1—O1^i^	91.39 (7)	C2—C1—H1B	109.1
O1—Co1—O1^i^	180.0	H1A—C1—H1B	107.9
O2^i^—Co1—N1	98.08 (6)	N1—C5—C6	108.58 (15)
O2—Co1—N1	81.92 (6)	N1—C5—H5A	110.0
O1—Co1—N1	78.83 (6)	C6—C5—H5A	110.0
O1^i^—Co1—N1	101.17 (6)	N1—C5—H5B	110.0
O2^i^—Co1—N1^i^	81.92 (6)	C6—C5—H5B	110.0
O2—Co1—N1^i^	98.08 (6)	H5A—C5—H5B	108.4
O1—Co1—N1^i^	101.17 (6)	O1—C6—N3	122.53 (19)
O1^i^—Co1—N1^i^	78.83 (6)	O1—C6—C5	119.19 (17)
N1—Co1—N1^i^	180.00 (8)	N3—C6—C5	118.28 (17)
C6—O1—Co1	113.26 (12)	N1—C3—C4	115.74 (16)
C3—N1—C5	113.47 (15)	N1—C3—H3A	108.3
C3—N1—C1	112.51 (15)	C4—C3—H3A	108.3
C5—N1—C1	111.69 (16)	N1—C3—H3B	108.3
C3—N1—Co1	107.54 (11)	C4—C3—H3B	108.3
C5—N1—Co1	103.12 (11)	H3A—C3—H3B	107.4
C1—N1—Co1	107.83 (11)	C6—N3—H3C	120.0
C2—O2—Co1	115.18 (12)	C6—N3—H3D	120.0
C2—N2—H2A	120.0	H3C—N3—H3D	120.0
C2—N2—H2B	120.0	C4—N4—H4A	120.0
H2A—N2—H2B	120.0	C4—N4—H4B	120.0
O2—C2—N2	121.59 (17)	H4A—N4—H4B	120.0
O2—C2—C1	121.70 (17)	O4—N5—O6	120.7 (2)
N2—C2—C1	116.70 (16)	O4—N5—O5	119.5 (2)
O3—C4—N4	124.06 (18)	O6—N5—O5	119.7 (2)
O3—C4—C3	121.43 (17)	H8A—O8—H8B	108 (2)
N4—C4—C3	114.46 (18)	H7A—O7—H7B	102 (2)
			
O2^i^—Co1—O1—C6	−81.44 (15)	Co1—O2—C2—N2	169.65 (15)
O2—Co1—O1—C6	98.56 (15)	Co1—O2—C2—C1	−11.4 (2)
N1—Co1—O1—C6	16.52 (14)	C3—N1—C1—C2	−118.18 (18)
N1^i^—Co1—O1—C6	−163.48 (14)	C5—N1—C1—C2	112.86 (18)
O2^i^—Co1—N1—C3	−62.62 (13)	Co1—N1—C1—C2	0.2 (2)
O2—Co1—N1—C3	117.38 (13)	O2—C2—C1—N1	7.4 (3)
O1—Co1—N1—C3	−152.44 (13)	N2—C2—C1—N1	−173.64 (17)
O1^i^—Co1—N1—C3	27.56 (13)	C3—N1—C5—C6	158.56 (16)
O2^i^—Co1—N1—C5	57.55 (12)	C1—N1—C5—C6	−72.98 (19)
O2—Co1—N1—C5	−122.45 (12)	Co1—N1—C5—C6	42.55 (17)
O1—Co1—N1—C5	−32.27 (12)	Co1—O1—C6—N3	−175.96 (18)
O1^i^—Co1—N1—C5	147.73 (12)	Co1—O1—C6—C5	4.6 (2)
O2^i^—Co1—N1—C1	175.82 (12)	N1—C5—C6—O1	−34.3 (3)
O2—Co1—N1—C1	−4.18 (12)	N1—C5—C6—N3	146.2 (2)
O1—Co1—N1—C1	86.00 (13)	C5—N1—C3—C4	68.0 (2)
O1^i^—Co1—N1—C1	−94.00 (13)	C1—N1—C3—C4	−60.0 (2)
O1—Co1—O2—C2	−70.37 (14)	Co1—N1—C3—C4	−178.61 (13)
O1^i^—Co1—O2—C2	109.63 (14)	O3—C4—C3—N1	−25.6 (3)
N1—Co1—O2—C2	8.54 (14)	N4—C4—C3—N1	156.74 (18)
N1^i^—Co1—O2—C2	−171.46 (14)		

Symmetry code: (i) −*x*+2, −*y*+1, −*z*+2.

### Hydrogen-bond geometry (Å, º)

**Table:**

*D*—H···*A*	*D*—H	H···*A*	*D*···*A*	*D*—H···*A*
N2—H2*A*···O6^ii^	0.86	2.26	3.026 (3)	149
N2—H2*B*···O3^iii^	0.86	1.96	2.816 (2)	173
N3—H3*C*···O8^iv^	0.86	2.11	2.958 (3)	168
N3—H3*D*···O5^iii^	0.86	2.16	3.001 (3)	166
N4—H4*A*···O7^v^	0.86	2.20	2.992 (3)	152
N4—H4*B*···O7	0.86	2.30	3.045 (3)	145
O7—H7*A*···O6	0.88 (2)	2.05 (2)	2.877 (3)	156 (3)
O7—H7*B*···O8^vi^	0.87 (2)	1.96 (2)	2.826 (3)	176 (3)
O8—H8*A*···O1^ii^	0.85 (2)	2.14 (2)	2.976 (2)	166 (3)
O8—H8*B*···O6	0.85 (2)	2.16 (2)	2.980 (3)	161 (3)
O8—H8*B*···O5	0.85 (2)	2.42 (3)	3.078 (3)	134 (3)

Symmetry codes: (ii) −*x*+1, −*y*+1, −*z*+2; (iii) −*x*+1, −*y*, −*z*+2; (iv) *x*+1, *y*, *z*+1; (v) −*x*+1, −*y*, −*z*+1; (vi) −*x*+1, −*y*+1, −*z*+1.
